# The Effect of Glycerol Supplements on Aerobic and Anaerobic Performance of Athletes and Sedentary Subjects

**DOI:** 10.2478/v10078-012-0065-x

**Published:** 2012-10-23

**Authors:** Suleyman Patlar, Hasan Yalçin, Ekrem Boyali

**Affiliations:** 1 Selcuk University, Physical Education and Sport High School, Konya, Turkey.; 2 Erciyes University, Engineering Faculty, Food Engineering Dep., Kayseri, Turkey.

**Keywords:** Glycerol, aerobic capacity, anaerobic power, athletes, sedentary subjects

## Abstract

The purpose of this study was to evaluate the effect of glycerol supplementation on aerobic and anaerobic exercise performance in sedentary subjects and athletes. The glycerol supplement treatments were as follows: 40 volunteers were selected and divided into two groups, sedentary and exercise groups. These two groups were further subdivided into two groups. The first group, the placebo (S), only consumed water; the second group (GS) consumed glycerol followed by water. Neither of these groups did any exercise for 20 days. The third and fourth groups consisted of the exercise group subjects; they were required to perform a 20-m shuttle run test every day for 20 days. The third group’s subjects, the placebo (E), only consumed water. The last group (GE) consumed glycerol followed by water. The Astrand Cycle Ergometer Test (ACET) was performed, and the Cosmed K4b^2^ portable gas analysis system was used to determine the aerobic capacity, while the Wingate Anaerobic Power Test (WAPT) was performed to determine the level of anaerobic power. The 20 Meter Shuttle Run Test (20MSRT) was performed after glycerol supplementation throughout the 20 days, and the exercise periods and distances were recorded.

The glycerol supplement was found to have an increasing effect on aerobic and anaerobic performance in GS, E and GE. A similar effect was found for the covered distances and time in the same groups. However, an adverse effect was found on body weight.

## Introduction

Glycerol (1,2,3-propanetriol) is produced from glucose, proteins, pyruvate, triacylglycerols and other glycerolipid metabolic pathways and it is a junctional metabolite in numerous pathways (Brisson et al., 2001). In particular, the metabolic importance of glycerol is based on the deprivation of glucose under aerobic and anaerobic conditions (Brisson et al., 2001). In humans, gluconeogenesis, glucose biosynthesis from non-carbohydrate precursors, mainly occurs in the liver and kidneys. While under normal health and dietary conditions, gluconeogenesis from glycerol accounts for less than 5% of glucose production; however, it appears that, after 62–86 hours of starvation, more than 20% of such production is derived from glycerol metabolism (Baba et al., 1995). During prolonged fasting, glycerol is the only source for gluconeogenesis, since glycogen reserves are depleted within two fasting days (Baba et al., 1995). This capacity to divert glycerol turnover into glucose production is an important evolutionary adaptation and allows for survival during undesirable conditions (Yeh et al., 1995).

Glycerol is a safe agent that does not approach toxic levels when administered orally in doses of <5 g/kg body weight. Glycerol, which can be ingested in comparatively large amounts, accumulates in body fluids, except for those of the brain and eyes, increasing osmotic pressure and the total volume of water in the body. Glycerol could be used as an energy substrate in nutrition, and could significantly contribute to the energy yield during exercise (Burelle et al., 2001). Considering its energy substrate function, glycerol could efficiently improve athletic performance (Montner et al., 1999). Glycerol’s osmoprotective solute quality can be used to improve physical endurance. The reduction in serum blood osmolality and the effects on ionic gradients caused by glycerol ingestion delay fatigue; therefore, endurance and athletic performance are improved (Hitchins et al., 1999). For a long time, the combined ingestion of glycerol and liquid has been used to increase body water volume, thus maintaining hydration by reducing the kidney’s’ water elimination rate. Glycerol could, therefore, play a very important role in thermoregulation, resistance to high temperatures and endurance in physical activities (Robergs and Griffin, 1998; Wagner, 1999).

When consumed orally, glycerol is rapidly absorbed and distributed between body fluid compartments before being slowly metabolized via the liver and kidneys. When consumed in combination with a substantial fluid intake, the osmotic pressure enhances the retention of this fluid and the expansion of various body fluid spaces. Typically, this allows fluid expansion or retention by reducing the urinary volume (Burge et al., 1993; Kavouras et al., 2006). Montner et al. (1999) reported that this fluid retention volume is in the range of 300–730 ml. Robergs and Griffin (1998) also reported that the use of glycerol increases blood osmolality and, when accompanied by large amounts of water (1500–2000 ml, or 26 ml/kg body weight), provides an osmotic drive that augments the retention of large quantities of water, which would otherwise be eliminated by the kidneys.

Glycerol, a naturally occurring metabolite, has been shown to be a safe and effective hyperhydrating agent (Magal et al., 2003). Glycerol combined with water hyperhydration increases total body water when compared with water hyperhydration alone. Different authors have shown conflicting results when assessing the effect of pre-exercise glycerol administration on subsequent performance functions. Several researchers have shown positive effects on performance after glycerol ingestion (Hitchins et al., 1999; Kavouras et al., 2006; Anderson et al., 2001; Coutts et al., 2002; Montner et al., 1996). For example, it has been suggested that glycerol-induced hyperhydration increases exercise performance (Burelle et al., 2001; Anderson et al., 2001; Coutts et al., 2002; Sawka and Montain 2000). Similarly, it has been demonstrated that glycerol ingestion increases, exercise tolerance in terms of time by approximately 24%. Glycerol ingestion increases the length of time that can be spent exercising because of the improvement in physical endurance. In addition, heart rate during exercise appears to be significantly lower after glycerol intake (Montner et al., 1996). Despite these findings, others have shown no benefits from pre-exercise hyperhydration with glycerol compared with hyperhydration with water alone (Pense and Turnagöl, 2011; Magal et al., 2003; Greiwe et al., 1998; Inder et al., 1998). Total body glycerol disposal can be divided into oxidation and gluconeogenesis. Most of the glycerol is turned into glucose in the liver by gluconeogenesis and the remainder is oxidized. The glucose produced is circulated in the blood stream and what is not required is converted into glycogen in the liver and muscles. Muscle glucose decomposes into pyruvic acid which supplies energy for exercise (Robergs and Griffin, 1998).

Burelle et al. (2001) reported that glycerol could significantly contribute to the energy yield, but the possible beneficial effect of glycerol ingestion on endurance performance remains a matter of debate. Although it seems that glycerol increases endurance, no studies have yet been able to determine if it generates significant improvement in physical performances. Wingo et al. (2004) examined the effect of the availability of glycerol drinks during exercise on the performance of athletes and they reported that future research should be conducted with pre-exercise glycerol supplementation, but not during exercise supplementation. A few studies have investigated the effect of glycerol on aerobic and anaerobic performance. However, no studies have investigated the effects of glycerol on the performance of soccer players. Therefore, the purpose of this study was to evaluate the effectiveness of glycerol on aerobic and anaerobic performance of both soccer players and sedentary people.

Although the effects of glycerol ingestion on sports performance are quite equivocal, glycerol has been reported to be a masking agent in the 2010 WADA Prohibited List (WADA). The reasons for this prohibition might be the potential effect of glycerol in terms of increasing the plasma volume and its damaging effects on renal functions. Glycerol has been recognized as a doping agent by the WADA in 2010 and should not be used as an ergogenic aid in competitive sports. However, this study was conducted before the WADA’s prohibition of glycerol.

## Material and Methods

### Subjects

Forty male volunteers participated in the study after completing health and medical screening questionnaires. The subjects were fully informed of all aspects of the study and signed statements of informed consent. Twenty subjects belonging to the exercise group were students of the Physical Education and Sport School of Higher Education. They were soccer players, members of the university’s soccer team. The other twenty subjects belonged to the sedentary group and were students of different faculties. The average age of the subjects was 22.82 ± 1.49 years.

### Procedure

Forty volunteers were selected and divided into two groups, sedentary and exercise groups. These two groups were further divided into two subgroups. The first group of ten subjects, the placebo group, consumed only water (26 ml/kg body weight) and is referred to as the sedentary group (S). The second group of ten subjects consumed glycerol (1.2 g/kg body weight) followed by water (26 ml/kg body weight) and is referred to as the glycerol supplemented sedentary group (GS). They did not exercise throughout the 20 days. The third and fourth groups, which comprised the exercise subgroups, performed a 20-m shuttle run test every day for 20 days. The third group’s subjects, the placebo (E), only consumed water (26 ml/kg body weight). The fourth group (GE) consumed glycerol (1.2 g/kg body weight) followed by water (26 ml/kg body weight).

The subjects were first familiarized with the exercise equipment. Throughtout the testing, exercise was performed on a cycle ergometer (Monark 814-E); ambient conditions were maintained at 30°C, the altitude was 1060 m and the barometrical pressure was 668 mm-Hg. The Astrand Cycle Ergometer Test (ACET) was performed, while the Cosmed K4b^2^ portable gas analysis system was used to determine aerobic capacity and the Wingate Anaerobic Power Test (WAPT) was performed to determine anaerobic power.

None of the subjects had consumed glycerol before participating in the study, to ensure that the subjects were blind to a pre-exercise fluid treatment. Throughout the study the subjects maintained similar eating habits and abstained from consuming alcohol, nicotine and caffeine. The subjects wore only lightweight shorts and were weighed. Body mass was determined before and after the experiment for each group. The subjects had a standard breakfast at 08.00 and ingested glycerol or water at 11.00. The E and GE groups exercised three hours after consuming the breakfast and liquid.

### Astrand Cycle Ergometer Test (ACET)

The ACET treatments were as follows: the cycle ergometer was calibrated and heart rate monitoring and timing equipment were provided to subjects after verifying that they functioned correctly. The subjects were weighed barefoot wearing lightweight shorts. They were hooked up to heart rate monitors and it was ensured that an adequate signal could be generated. The subjects’ resting heart rates were recorded. Bicycle seats and handle bars were adjusted to suit individual subjects.

Following a warm-up at a low intensity the test commenced with a workload of 900 kpm/min (150W) and the heart rate was recorded each minute. The last 15 seconds of each minute (×4) was used to record the value for that minute. If the heart rate of the subject was <120 bpm after two minutes of exercise, the work load was increased by 150 to 300 kpm/min (25–50W). The subjects were required to exercise for a minimum of six minutes at the final work load. If the difference between the fifth- and sixth-minute heart rates was ≤5 bpm, the work load was reduced to a minimum for a cool-down period. However, if the heart rate difference was >5 bpm, the work load continued for another minute or more until two consecutive heart rates differed by no more than 5 bpm. The test did not last more than ten minutes. The test was terminated if the heart rate exceeded 170 bpm (or 85% of the predicted maximum heart rate). Max VO_2_ was determined using the Cosmed K4b^2^ portable gas analysis system. The expired air was measured and analyzed breath by breath using an automated online system (K4 B^2^ system, Cosmed Srl, Rome, Italy) and the heart rate was monitored and recorded throughout the test. Before each test, the device was calibrated according to the manufacturer’s instructions. The criteria to reach VO_2max_ were as follows: a plateau in oxygen uptake must occur as the workload is increasing, a respiratory exchange ratio must exceed 1.15 and the heart rate must be within ten beats of the age-predicted maximal heart rate calculated as 220 bpm−age. The subjects exercised at a minimal work load for the cool-down period for four minutes.

### Wingate Anaerobic Power Test (WAPT)

The testing device was a mechanically braked bicycle ergometer. Before the test, the subjects’ feet were firmly strapped to the pedals, and the seat height and handlebars were adjusted for optimal comfort and pedalling efficiency. During the rest period the subjects were instructed to perform the test with maximum intensity. The subjects began pedaling as fast as possible without any resistance after a five-minute warm up. Then the WAPT was initiated against minimal resistance. A fixed resistance was applied to the flywheel within three seconds, and the subjects continued to pedal “all out” for 30 seconds. A computer continuously recorded the flywheel revolutions in five-second intervals. The flywheel resistance was set at 0.075 kg per kg body weight. The average power was determined by measuring the power outputs observed during the 30 seconds of exercise on a laboratory cycle ergometer.

### 20 Meter Shuttle Run Test (20MSRT)

The subjects warmed up for several minutes by jogging followed by stretching. The test program was installed on the computer and initiated. A single beep was emitted at regular intervals. The subjects had to complete a lap or shuttle (foot on or over the line) with each beep. If the subjects completed a lap early they had to wait for the beep before starting the next lap. A triple beep indicated the start of a new level with a slightly faster speed required to complete each lap. The subjects were encouraged to complete as many levels as possible. An observer monitored the progress of a given subject, recording each completed lap on the recorder form. The subjects were instructed to turn by pivoting and not to run in a wide arc. The test was terminated when a subject was two or more steps from the line, for two consecutive laps. The observer alerted the subject at this time. At the end of the 20MSRT the subjects continued walking for several minutes, followed by stretching exercises upon completion of the test. Information was entered into the data entry screen.

This test (the 20MSRT) was performed after supplementation with glycerol throughout the 20 days, and the exercise durations and distances were recorded.

### Statistical Analysis

Descriptive statistics (mean ± SEM) were calculated for all the variables. The one-way analysis of variance (ANOVA) and Duncan’s Multiple Range Test were used to assess the differences between the groups (S, GS, E and GE). The differences within each group were determined with the Related T Test. Significance was considered for all analyses at the level p < 0.05. All of the statistical analyses were performed using the Statistical Package for the Social Sciences (SPSS Inc., Chicago, IL, USA).

## Results

**Figure 1:**
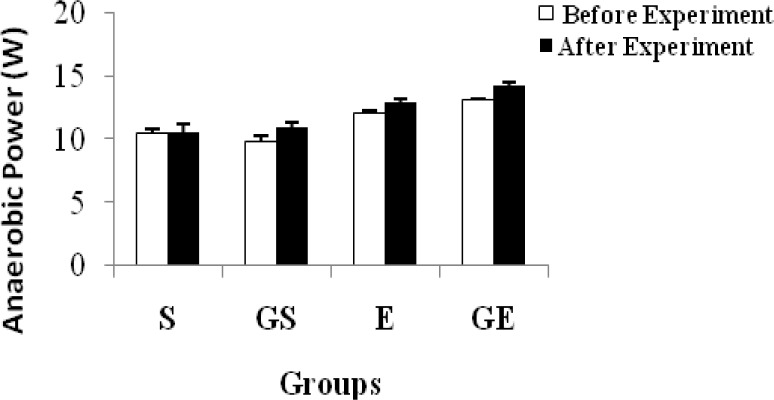
Changes in anaerobic power of subjects (mean ±SE)

**Figure 2:**
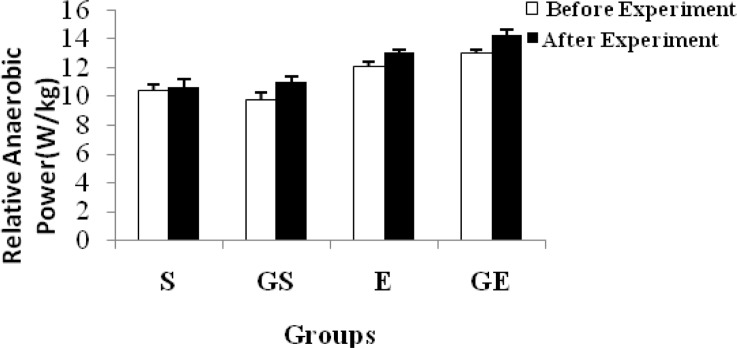
Changes in relative anaerobic power of subjects (mean ±SE)

The anaerobic power of each group is shown in Table 1 and Figure 1. The anaerobic power was determined as average power. Although a significant difference (p < 0.05) was not shown in S before and after the experiment, it was observed in GS, E and GE. The relative anaerobic power shown in Table 2 and Figure 2 was obtained by dividing the anaerobic power by the body weight of the subjects. A similar result was obtained in this way.

**Figure 3:**
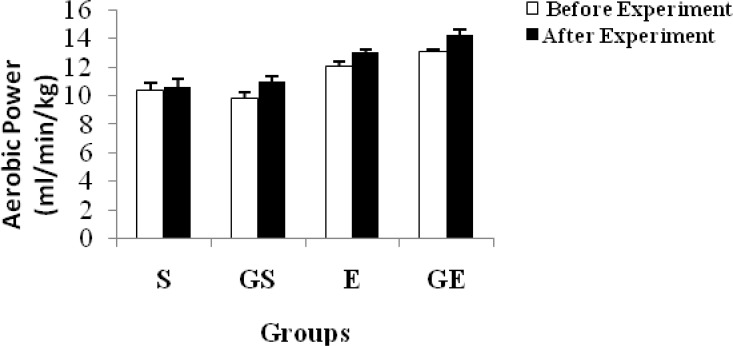
Changes in aerobic capacity of subjects (mean ±SE)

The aerobic power of each group is shown in Table 3 and Figure 3. A significant difference (p < 0.05) was seen in each group, and the results were compared before and after the experiment after glycerol was consumed, except in S. Table 3 shows that glycerol has an increasing effect on aerobic power.

**Figure 4:**
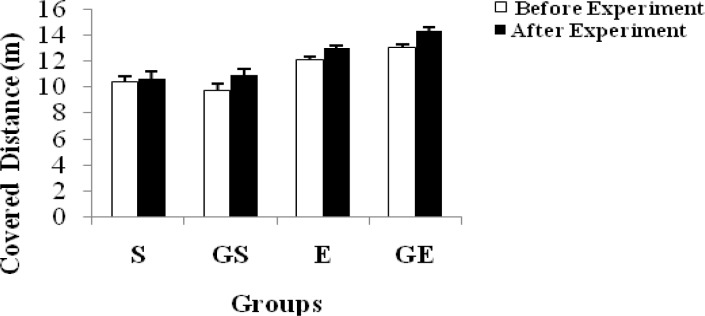
Changes in covered distance of subjects (mean ±SE)

**Figure 5:**
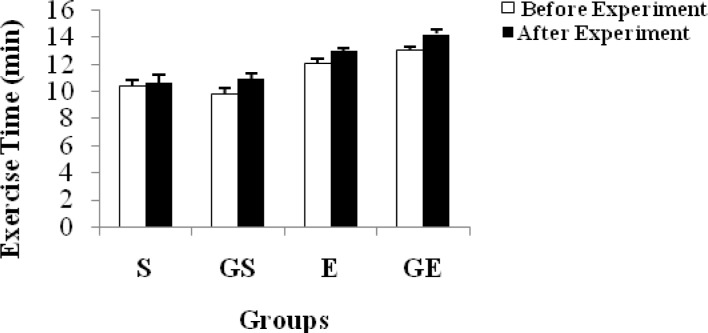
Changes in exercise time of subjects (mean ±SE)

**Figure 6:**
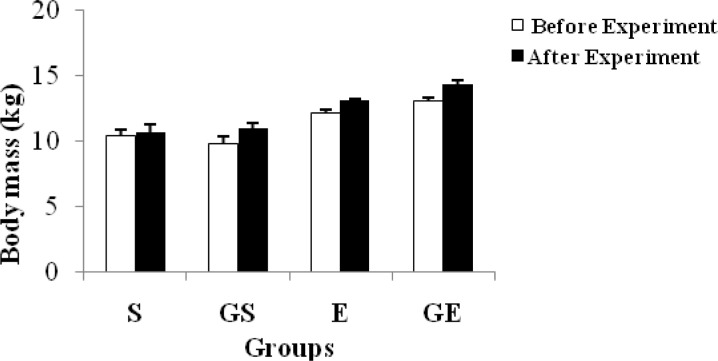
Changes in body mass of subjects (mean ±SE)

The distance covered in the shuttle run and time of each group are shown in Table 4 and Figure 4 and Table 5 and Figure 5, respectively. In addition, changes in body mass of the subjects are shown in Table 6 and Figure 6.

## Discussion

According to this table, it is clear that glycerol has an increasing effect on anaerobic power, because the GS and GE groups were given glycerol. The difference in E may be based on both the fact the subjects exercised for 20 days and the training status of the subjects. A significant difference was observed between E and GE after the experiment. Furthermore, a similar difference was seen between S and GS after the experiment. These differences may be due to the effects of glycerol supplementation. There is no literature that examines the effect of glycerol on the anaerobic performance of athletes and sedentary subjects. In humans, glycerol allows ATP production through gluconeogenesis. Glycerol is a gluconeogenic substance, which can be metabolized in sufficient time to provide energy during intensive exercise (Kavouras et al., 1998).

The highest aerobic power was obtained in GE and the lowest value was registered in S after the experiment. GE’s aerobic power was higher than that of E (p < 0.05) but GS’s aerobic power was not, compared with that of S before and after the experiment. The aerobic power was found to be 47.66 ± 1.62 and 51.71 ± 1.36 ml/min/kg (p < 0.05) in GS before and after the experiment, respectively. The glycerol effect was clearly seen in this group. Moreover, a similar effect was found between GE and E. However, the aerobic power was equal to 51.71 ± 1.36 and 50.57 ± 1.88 ml/min/kg (p < 0.05) in GS and S, respectively, after the experiment. No statistically significant difference was found between the groups. Sawka et al. (2001) reported that hypohydration decreases maximal aerobic power. Hypohydration results in larger decrements in the capacity for aerobic exercise. One hyperhydrating strategy that has recently received attention as a possible means of reducing hypohydration is the use of glycerol.

Glycerol ingestion increases fluid retention by reducing free water (Latzka et al., 1997). Hence glycerol hyperhydration improves exercise performance (Hitchins et al., 1999; Anderson et al., 2001; Coutts et al., 2002). In an effort to explain the performance improvements caused by glycerol, Hitchins et al. (1999) proposed that the reductions in serum blood osmolality or electrolytes and their consequent effect on ionic gradients across cells induced by glycerol hyperhydration may delay the onset of either muscle or central fatigue. Therefore, the increase in aerobic performance after glycerol supplementation in this study is meaningful.

According to Table 4 and Figure 4 similar significant differences are seen in aerobic performance and are shown in each group except S, when comparing that variable before and after the experiment. The differences in GS and GE may be due to glycerol and the difference in E may be due to the training status of the subjects. Moreover, a difference was observed in the binary exercise and sedentary groups. The training status of the subjects might have caused this difference. The highest and the lowest distances covered were obtained in GE and S, respectively. According to this table, glycerol has an increasing effect on the distance covered during the run. Wagner (1999) suggested that glycerol hyperhydration might be most effective for those competing in ultra-distance sports. This suggestion should be the focus of future studies.

The exercise time (Table 5 and Figure 5) was found to be significantly different (p < 0.05) in each group taking glycerol when compared to the status before and after the experiment, except in S. There was a significant difference between each group after the experiment. However, there was no difference between S and GS after the experiment. A significant difference was seen between the sedentary and exercise groups before the experiment. This difference may be based on the training status of the subjects. Glycerol ingestion improved the time trial performance compared with the placebo treatment in this study. An early study by Montner et al. (1996) showed a 21–24% improvement in endurance time when exercise was performed at a workload equivalent to 60% of maximum power output following pre-exercise glycerol hyperhydration. Both Hitchins et al. (1999) and Anderson et al. (2001) reported a 5% improvement in a 30-minute and a 15-minute cycling time trial, respectively, following glycerol hyperhydration.

Latzka et al. (1998) had subjects exercise to exhaustion during uncompensable exercise-heat stress. They found that glycerol hyperhydration extended the endurance time from 30 to 34 minutes. Furthermore, Coutts et al. (2002) demonstrated a reduced decrement in performance time following glycerol hyperhydration compared with the ingestion of a placebo fluid. Scheett et al. (2001) reported a significantly longer time taken to reach exhaustion (12.6%) with subjects who had taken glycerol compared with those who had had a water solution during exercise. Seifert et al. (1995) recorded an 8% improvement in the time taken to complete a 600-revolution time trial (355 versus 385). Kavouras et al. (1998) found that the cycling time taken to reach exhaustion in individuals subjected to a pre-exercise dehydration protocol was longest after a glycerol rehydration protocol compared to the after-water and no-fluid rehydration protocols.

In many of the studies, the subjects were weighed before and after exercise each day due to water loss. However, in this study, subjects were weighed at the beginning and, after 20 days of the experiment to obtain changes in body mass. There was a significant decrease (p < 0.05) in E and GE, whereas there was a significant increase in body weight in GS. Glycerol supplementation may be the reason for the gain in body weight in GS. Moreover, this group was untrained which may be the second reason for the gain in body mass. The significant decrease in body mass in E and GE may be due to exercise.

Many of the responses are highly dependent on a multitude of interacting factors. These factors include the pre-test hydration status, the acclimation and training status of the participants, the performance environment and the exercise intensity coupled with the performance time. The timing of the exercise performance after the hydration phase also appears to be a major factor.

## Conclusion

This study determined the impact of glycerol when comparisons were made between groups with glycerol supplementation and groups that had received placebo as a pre-exercise strategy. Glycerol was found to influence exercise performance of the soccer players. Furthermore, this study demonstrated that glycerol can be used as an ergogenic aid for soccer players when its effects were compared with those of the placebo groups. However, at present, it must be noted that glycerol is not allowed to be used in competitive sport. Otherwise, indicators should be made for amateur athletes and these exercising recreationally.

## Figures and Tables

**Table 1:** Mean values of anaerobic power

**Group**	**Anaerobic Power(W)**	
	**Before Experiment**	**After Experiment**
**S**	482,82 ± 12.37 Ba	502.92 ± 14.98 Da
**GS**	490,81 ± 17.21 Bb	537.99 ± 13.34 Ca
**E**	507,85 ± 15.16 Bb	571.02 ± 9.49 Ba
**GE**	581,89 ± 17.87 Ab	621.60 ± 14.42 Aa

abc: Different letters indicate a significant difference between rows(p<0.05).

ABCD: Different letters indicate a significant difference between columns (p<0.05).

**Table 2:** Mean values of relative anaerobic power

**Group**	**Relative Anaerobic Power (W/kg)**	
	**Before Experiment**	**After Experiment**
**S**	6.78 ± 0.26 Ba	7.13 ± 0.46 Ba
**GS**	6.57 ± 0.23 Bb	7.05 ± 0.20 Ba
**E**	6.78 ± 0.22 Bb	7.78 ± 0.27 Ba
**GE**	8.28 ± 0.25 Ab	9.08 ± 0.17 Aa

ab: Different letters indicate a significant difference between rows(p<0.05).

AB: Different letters indicate a significant difference between columns (p<0.05).

**Table 3:** Mean values of aerobic capacity

**Group**	**Aerobic Power (ml/min/kg)**	
	**Before Experiment**	**After Experiment**
**S**	49.67 ± 1.56 Ca	50.57 ±1.88 Ca
**GS**	47.66 ± 1.62 Cb	51.71 ± 1.36 Ca
**E**	55.34 ± 0.81 Bb	58.60 ± 0.59 Ba
**GE**	58.94 ± 0.70 Ab	62.64 ± 1.11 Aa

abc: Different letters indicate a significant difference between rows(p<0.05).

ABC: Different letters indicate a significant difference between columns (p<0.05).

**Table 4:** Mean values of covered distance

**Group**	**Covered Distance (m)**	
	**Before Experiment**	**After Experiment**
**S**	1860 ± 101.11 Ba	1926 ± 123.54 Ba
**GS**	1730 ± 105.04 Bb	1992 ± 91.66 Ba
**E**	2232 ± 60.16 Ab	2474 ± 97.62 Aa
**GE**	2392 ± 104.70 Ab	2674 ± 147.85 Aa

ab: Different letters indicate a significant difference between rows(p<0.05).

AB: Different letters indicate a significant difference between columns (p<0.05).

**Table 5:** Mean values of exercise time

**Group**	**Exercise Time (min)**	
	**Before Experiment**	**After Experiment**
**S**	10.40 ± 0.46 Ba	10.65 ± 0.55 Ca
**GS**	9.78 ± 0.50 Bb	10.96 ± 0.41 Ca
**E**	12.10 ± 0.27 Ab	13.03 ± 0.19 Ba
**GE**	13.06 ± 0.20 Ab	14.27 ± 0.35 Aa

abc: Different letters indicate a significant difference between rows(p<0.05).

ABC: Different letters indicate a significant difference between columns (p<0.05).

**Table 6:** Mean values of body mass

**Group**	**Body Weight (kg)**	
	**Before Experiment**	**After Experiment**
**S**	72,15 ± 3.16 Aa	72,25 ± 3.14 ABa
**GS**	75,20 ± 3.09 Ab	76,80 ± 3.11 Aa
**E**	75,15 ± 2.37 Aa	73,92 ± 2.21 ABb
**GE**	70,40 ± 1.61 Aa	68,50 ± 1.53 Bb

ab: Different letters indicate a significant difference between rows(p<0.05).

AB: Different letters indicate a significant difference between columns (p<0.05).
